# Alterations of Lysine Acetylation Profile in Murine Skeletal Muscles Upon Exercise

**DOI:** 10.3389/fnagi.2022.859313

**Published:** 2022-05-03

**Authors:** Dehuan Liang, Cheng Chen, Song Huang, Sujuan Liu, Li Fu, Yanmei Niu

**Affiliations:** ^1^Department of Rehabilitation, School of Medical Technology, Tianjin Medical University, Tianjin, China; ^2^Department of Anatomy and Histology, School of Basic Medical Science, Tianjin Medical University, Tianjin, China

**Keywords:** aerobic exercise, bioinformatics analysis, lysine acetylation, muscle, proteins

## Abstract

**Objective:**

Regular exercise is a powerful tool that enhances skeletal muscle mass and strength. Lysine acetylation is an important post-translational modification (PTM) involved in a broad array of cellular functions. Skeletal muscle protein contains a considerable number of lysine-acetylated (Kac) sites, so we aimed to investigate the effects of exercise-induced lysine acetylation on skeletal muscle proteins.

**Methods:**

We randomly divided 20 male C57BL/6 mice into exercise and control groups. After 6 weeks of treadmill exercise, a lysine acetylation proteomics analysis of the gastrocnemius muscles of mice was performed.

**Results:**

A total of 2,254 lysine acetylation sites in 693 protein groups were identified, among which 1,916 sites in 528 proteins were quantified. The enrichment analysis suggested that protein acetylation could influence both structural and functional muscle protein properties. Moreover, molecular docking revealed that mimicking protein deacetylation primarily influenced the interaction between substrates and enzymes.

**Conclusion:**

Exercise-induced lysine acetylation appears to be a crucial contributor to the alteration of skeletal muscle protein binding free energy, suggesting that its modulation is a potential approach for improving exercise performance.

## Introduction

Protein lysine acetylation is an indispensable and conserved post-translational modification (PTM) that occurs in prokaryotic and eukaryotic cells (Xing and Poirier, 2012). Acetylation is involved in energy metabolism, gene expression, and cell signaling in both animals and bacteria (Xing and Poirier, 2012; Fukushima and Lopaschuk, 2016; Narita et al., 2019). In recent years, many studies have reported that lysine acetylation regulates a wide range of cellular functions in skeletal muscle (Lundby et al., 2012; Moresi et al., 2015; Tsuda et al., 2018). For example, histone acetylation in the nucleus controls MEF2- and MyoD-dependent transcription, which regulates myoblast differentiation and muscle homeostasis (Moresi et al., 2015). Tsuda found that protein acetylation in the skeletal muscle mitochondria of mice was linked with intermediary metabolism (Tsuda et al., 2018). In addition, Lundby discovered a mass of lysine acetylation sites in 16 different tissues of rats, where the degree of lysine acetylation of muscle was highest (Lundby et al., 2012). Furthermore, they found that proteins involved in muscle contraction and enzymes generating ATP were all acetylated (Lundby et al., 2012), which provided a new and important clue that acetylation modification regulates the energy and metabolism of muscle.

The skeletal muscle is an organ for the maintenance of mobility, and exercise training in turn is known to positively affect the skeletal muscle system (Widegren et al., 2001; Russell et al., 2014; Nelson et al., 2020). Cellular stress triggered by exercise can alter the coordinated activity of acetyltransferases and deacetylases to regulate lysine acetylation (Marton et al., 2010; Menzies and Auwerx, 2013). Previous studies have found dynamic changes in histone acetylation and mitochondrial protein acetylation in the skeletal muscle during exercise (McGee et al., 2009; Overmyer et al., 2015). In our previous study, aerobic training or Scriptaid dramatically improved the exercise performance of mice, including endurance, grip strength, and citrate synthase activity (Huang et al., 2020). These changes were also accompanied by an increase in titin lysine acetylation in gastrocnemius tissue, which was postulated to function as a mediator to regulate muscle protein function at the molecular level (Huang et al., 2020). Given the important role of skeletal muscle in exercise or physical activities (Gan et al., 2018), we speculate that exercise-induced lysine acetylation in muscle may be an important factor mediating exercise performance.

The purpose of this study was to profile protein lysine acetylation in the skeletal muscle of exercise and control groups using different proteomic technologies and bioinformatics tools. Our preliminary data demonstrated widespread protein lysine acetylation in the skeletal muscle of mice after exercise. Subsequent proteomic studies confirmed that the different acetylation patterns of muscle proteins were closely related to their structure and function of muscle. Then, molecular docking data suggested that lysine acetylation modification could change the binding of substrates and enzymes, ultimately influencing enzyme activity and energy transformation. Taken together, exercise-induced lysine acetylation might play an important role in muscle structural and functional proteins properties, indicating the crucial role of protein acetylation in skeletal muscle physiology.

## Materials and Methods

### Animals

Twenty 5-week-old male C57BL/6 mice were purchased from Beijing HFK Bioscience Co., (Beijing, China) in this study. The animal study was approved by the Tianjin Medical University Animal Care and Use Committee (approval number: SYXK-2019-0004) under the guidelines of the Chinese Academy of Sciences. The mice were housed in a temperature-controlled room (22 ± 2°C) with a 12-h light/dark cycle (light on from 08:00 to 20:00) and free access to food and water. All animals were allowed to acclimatize to the laboratory environment for 1 week.

At the beginning of the experiment, the mice were randomly divided into two groups: a sedentary control group (*n* = 10) and an exercise group (*n* = 10). Mice in the control group were kept sedentary for 6 weeks. Mice in the exercise group were familiarized with the treadmill 5 days before the experiment by exercising them for 1 h at 8 m/min; each day thereafter, the speed was increased by 2 m/min until the speed has reached 12 m/min. Then, mice in the exercise group underwent 6 weeks of treadmill exercise for 5 days per week (1 h/day) on a 0% grade at the intensity of 75% VO2max (12 m/min) as previously described (Fernando et al., 1993). During the 1-h session, the mice ran on the treadmill continuously without rest intervals throughout the whole 60-min time. By the end of the 6-week exercise, the body weight of the mice was determined with an ImpediVET analyzer (ImpediMed). Forty-eight hours after the last bout of exercise, mice were fasted for 14 h and then anesthetized with isoflurane. The gastrocnemius was dissected and weighed, frozen in liquid nitrogen, and then stored at −80°C for later analysis.

### Protein Extraction, Trypsin Digestion, and TMT Labeling

Each skeletal muscle sample was ground with liquid nitrogen and transferred to a 5-mL centrifuge tube. Then, the samples were sonicated three times on ice using a high-intensity ultrasonic processor (Scientz, Ningbo, China) in lysis buffer [8 M urea, 10 mM dithiothreitol (DTT), 3 μM trichostatin (TSA), 50 mM nicotinamide (NAM), and 1% protease inhibitor cocktail], followed by centrifugation at 12,000 × *g* at 4°C for 10 min. After that, we discarded the supernatant and washed the remaining precipitate three times. The protein was redissolved in 8 M urea, and its concentration was determined with a BCA kit.

For trypsin digestion, 5 mM DTT was added to implement the reduction of proteins for 30 min at 56°C, and 11 mM iodoacetamide was added to perform alkylation of proteins for 15 min at room temperature in darkness. Then, the protein sample was diluted by adding 100 mM TEAB to a urea concentration of less than 2 M. In the first digestion, trypsin was added at a 1:50 trypsin-to-protein mass ratio, and this digestion was performed at 37°C overnight. For the second digestion, trypsin was added at a mass ratio of 1:100 trypsin/protein and digested for 4 h at 37°C.

After trypsin digestion, the peptide was desalted by a Strata X C18 SPE column (Phenomenex) and vacuum-dried. The peptide was reconstituted in 0.5 M TEAB and processed according to the manufacturer’s protocol for the TMT kit/iTRAQ kit. In brief, one unit of TMT/iTRAQ reagent was thawed and reconstituted in acetonitrile. The peptide mixtures were then incubated for 2 h at room temperature and pooled, desalted, and dried by vacuum centrifugation.

### HPLC Fractionation and Affinity Enrichment

The TMT-labeled samples were fractionated by high-pH reverse-phase HPLC using a Thermo Betasil C18 column (5 μm particles, 10 mm ID, and 250 mm length). In brief, peptides were first separated with a gradient of 8–32% acetonitrile (pH 9.0) over 60 min into 60 fractions. Finally, the peptides were combined and dried by vacuum centrifugation.

Immunoprecipitation was performed to enrich modified peptides. IP buffer solution (100 mM NaCl, 1 mM EDTA, 50 mM Tris–HCl, 0.5% NP-40, pH 8.0) was added to dissolve tryptic peptides. Then, the liquid supernatant was incubated with prewashed antibody beads (acetyl-lysine antibody beads lot number PTM1001, PTM Biolabs Inc., China) at 4°C overnight with gentle shaking. The beads were washed four times with IP buffer solution and two times with double-distilled H_2_O. Then, we eluted bound peptides with 0.1% trifluoroacetic acid (TFA) and vacuum-dried them. For LC-MS/MS analysis, the obtained peptides were desalted with C18 ZipTips (Millipore) in accordance with the manufacturer’s instructions.

### LC-MS/MS Analysis and Data Research

Peptides were dissolved in solvent A (0.1% formic acid) and directly loaded onto a homemade reversed-phase analytical column (15 cm length, 75 μm i.d.). The gradient comprised an increase from 6 to 23% solvent B (0.1% formic acid in 98% acetonitrile) over 26 min, from 23 to 35% for 8 min, increasing to 80% in 3 min and then holding at 80% for the last 3 min, all at a constant flow rate of 400 nL/min on an EASY-nLC 1000 UPLC system. The peptides were subjected to an NSI source followed by tandem mass spectrometry (MS/MS) in a Q Exactive™ Plus (Thermo) coupled online to the UPLC. The applied electrospray voltage was 2.0 kV. The m/z scan range was 350–1,800 for the full scan, and intact peptides were detected in the Orbitrap at a resolution of 70,000. The peptides were then selected for MS/MS using an NCE setting of 28. Ion fragments were detected in the Orbitrap at a resolution of 17,500 (m/z 200). A data-dependent procedure alternated between one MS scan followed by 20 MS/MS scans with 15.0 s dynamic exclusion. Automatic gain control was used to prevent overfilling of the ion trap, and 5e4 ions were accumulated for the generation of MS/MS spectra. The fixed first mass was set as 100 m/z.

Then, we processed the LC-MS/MS data with the MaxQuant search engine (v.1.5.2.8). Tandem mass spectra were searched against the UniProt database concatenated with the reverse decoy database. Trypsin/P was specified as the cleavage enzyme allowing up to four missing cleavages. In the first search and the main search, the mass tolerances for precursor ions were set as 20 and 5 ppm, respectively. Meanwhile, the mass tolerance for fragment ions was set as 0.02 Da. Carbamidomethylation on Cys was specified as a fixed modification, while acetylation modification and oxidation on Met were variable modifications. The false discovery rate (FDR) and the minimum score for modified peptides were adjusted to <1% and >40, respectively. The retrieval parameter settings and quality control of mass spectrometry are shown in Supplementary Material.

### Bioinformatics Analyses

For subsequent bioinformatics analysis, normalization with protein quantification to remove the effect of protein expression on modification abundance was performed. Protein acetylation was identified on the basis of the following criteria: *t*-test with *p*-value < 0.05, fold change > 1.2 or < 0.83, which were consistent with previous studies (Ren et al., 2013; Wu et al., 2016; Huang et al., 2019; Yu et al., 2021). For hierarchical clustering based on Kac sites of proteins associated with muscle function, we collated all the categories obtained after enrichment along with their *P*-values. The Gene Ontology (GO) annotation proteome was derived from the UniProt-GOA database,^1^ and the proteins were classified by GO annotation based on three categories: biological process, cellular component, and molecular function. For each category, a two-tailed Fisher’s exact test was employed to test the enrichment of the identified modified protein against all proteins from the species database. The GO terms with a corrected *p*-value < 0.05 were considered significant. All differentially expressed modified proteins were searched against the STRING database^2^ for protein–protein interactions (PPIs). Only interactions between the proteins showing acetylation variance were selected, thereby excluding external candidates. The PPI networks for the identified acetylated proteins were analyzed by Cytoscape 3.9.0 software using interaction data.

### Molecular Docking

AutoDock 4.2 software, an excellent tool to recognize docking affinity between individual ligands and specific proteins (Seeliger and de Groot, 2010; Rizvi et al., 2013), was utilized in all the docking experiments. Protein receptors were taken from UniProt^3^ in PDB format, and then, specific-site lysine-to-arginine (K-to-R) changes were made in PyMOL 2.5.1, mimicking deacetylated lysine residues. Ligands were obtained from PubChem^4^ in SDF format and converted to PDB format by Open Babel 3.1.1. After preparing proteins and ligands, we ran Autogrid to calculate the affinity maps, and the size of the grid box was kept large enough to at least allow the ligand to rotate freely. Then, the docking of the protein and ligand was performed. Grid and docking parameter files were given as GPF files and DPF files in AutoDock. Finally, we used Chimaera 1.15 and Notepad^++^ 7.9.5 to read the results.

### RNA Extraction and Quantitative Real-Time RT-PCR

Total RNA was extracted from gastrocnemius muscle and reverse-transcribed to cDNA using TransScript One-Step gDNA Removal and cDNA Synthesis SuperMix (TransGen Biotech). The synthesized cDNA was used for PCR with specific cycles. The primer sequences were as follows: MyHC I, 5′-GCCTGGGCTTA CCTCTCTATCAC-3′ (forward), 5′-CTTCTCAGACTTCCGC AGGAA-3′ (reverse); MyHC IIa, 5′-CAGCTGCACCTTCTCG TTTG-3′ (forward), 5′-CCCGAAAACGGCCATCT-3′ (reverse); MyHC IIb, 5′-CAATCAGGAACCTTCGGAACAC-3′ (forward), 5′-GTCCTGGCCTCTGAGAGCAT-3′ (reverse); MyHC IIx, 5′-G GACCCACGGTCGAAGTTG-3′ (forward), 5′-CCCGAAAACG GCCATCT-3′ (reverse); and GAPDH, 5′-GCACAGTCAAGGC CGAGAAT-3′ (forward), 5′-GCCTTCTCCATGGTGGTGAA-3′ (reverse).

PCR amplification was performed using the Roche LightCycler 480 real-time PCR system (Roche Life Science, United States) and the PerfectStart Uni RT-qPCR Kit (TransGen Biotech) according to the manufacturers’ instructions. The relative expression levels of the target genes were determined using the 2^–ΔΔCT^ method.

### Oil Red O Staining

Gastrocnemius samples were dissected and fixed for 24 h in 10% neutral buffered formalin. Then, we embedded tissues with paraffin and sliced them into 7-μm serial horizontal sections. The gastrocnemius muscle sections were stained with Oil Red O, and the cross-sectional area (CSA) of the muscle fiber was assessed under light microscopy (AF 6000, Leica). Five images per animal (*n* = 3) and at least 60 muscle cells per image were used in the statistical analysis (ImageJ software). The total average area was used to determine the CSA per square micrometer.

### Statistical Analysis

The data are presented as the means ± SEM, and the differences were analyzed using Student’s *t*-test or two-way ANOVA. All data were considered significantly different when *P* < 0.05. We used SPSS to make statistical comparisons.

## Results

### Identification of Lysine Acetylation Proteins and Sites in the Gastrocnemius Muscle

**FIGURE 1:**
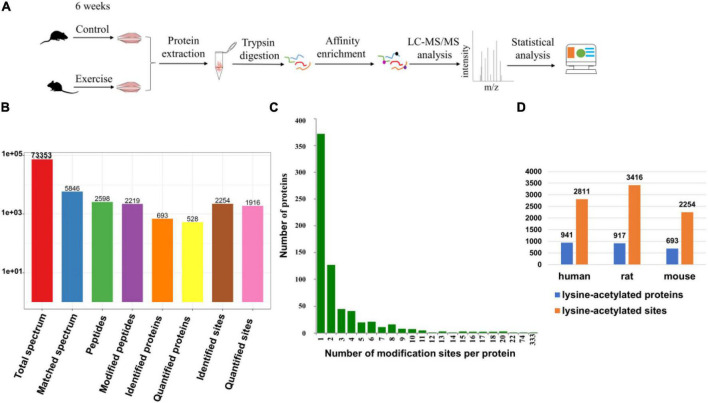
Identification of lysine-acetylated proteins and sites in the gastrocnemius muscle. **(A)** Schematic of the LC/MS-based quantitative acetylproteomic strategy. **(B)** The basic statistical figure for MS results. **(C)** Distribution of acetylated proteins based on the number of acetylated peptides. **(D)** Comparison of our proteomic study with Lundby’s (Lundby et al., 2012). The figure shows the number of lysine-acetylated proteins and sites identified in muscle from three different species.

In this study, gastrocnemius muscle samples from the two groups were mapped to identify the lysine acetylation sites of proteins, and the workflow of the experimental procedures is shown in Figure 1A. To verify the initial mass spectrometry data, we detected the lengths of all the obtained peptides and the quality errors. The lengths of most peptides, ranging between 7 and 20, matched the lengths of the tryptic peptides (Supplementary Figure 1), indicating that the sample preparation met the standard for proteomics analysis. The average mass error for all the identified peptides was nearly zero, and first-order mass errors were less than 10 ppm, meaning that the mass accuracy of the MS data met the requirement (Supplementary Figure 2). During the identification, a total of 73,353 secondary spectra were obtained by mass spectrometry. Then, we used mass spectrometry secondary spectra to search against protein theory data. The available efficiency was 5,846, the spectrum utilization rate was 8.0%, and the peptides were resolved into 2,598 peptides and 2,219 acetylated peptides. In addition, 2,254 acetylation sites were identified in 693 proteins, including 1,916 quantified sites in 528 quantified proteins (Figure 1B). Among these proteins, 372 proteins (53.70%) had one Kac site and 108 proteins (15.58%) contained five or more Kac sites (Figure 1C). There were 15 proteins (2.16%) that contained more than 15 Kac sites (Figure 1C). Interestingly, those proteins that contained more than 15 Kac sites were mainly distributed in sarcomeres, especially titin (333 sites) (Huang et al., 2020) and myosin-4 (74 sites). On average, there were 3.25 Kac modification sites per protein (Figure 1D) in the gastrocnemius of mice. In another independent experiment, Lundby et al. (2012) found 3,416 sites from 917 proteins in the rat muscle and 2,811 sites from 941 proteins in the human muscle, where the average numbers of acetylation sites per protein were 3.73 and 2.99, respectively (Figure 1D). Comparison analysis of these data of three species indicated that protein acetylation widely exists in the skeletal muscle of mice, rats, and humans.

### Effect of Aerobic Exercise on the Muscle of Mice

**FIGURE 2:**
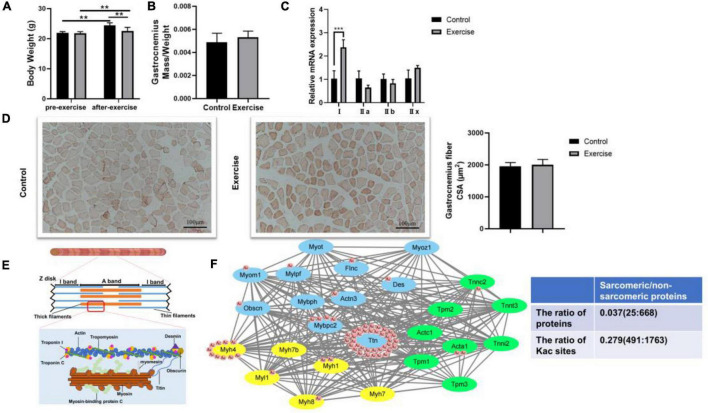
Effect of aerobic exercise on the muscle of mice. **(A)** Body weight of mice was examined 48 h after treadmill exercise. **P < 0.01. **(B)** Gastrocnemius muscle mass was measured and normalized to body weight. **(C)** The gastrocnemius muscle is mainly composed of MyHC IIb and MyHC IIx fiber. Compared with the control group, MyHC I was upregulated in the gastrocnemius of trained mice. Values are means ± SEM; *n* = 3 in each group; ***P < 0.001. **(D)** Representative Oil Red O staining of muscle sections and analysis of gastrocnemius fiber CSA in the control and exercise groups. **(E)** Schematic representation of muscle sarcomeres. Only the main acetylated/deacetylated proteins in the sarcomere and the basic contractile unit of skeletal muscle are shown. **(F)** Lysine sites of sarcomeric proteins were identified in the mouse skeletal muscle, and a protein–protein interaction network was visualized with STRING. Each green node represents proteins expressed in thin filaments, each yellow node represents proteins expressed in thick filaments, and each blue node represents sarcomere-associated proteins. Some nodes are surrounded by red circles, which represent lysine acetylation induced by exercise. The table shows the comparison of sarcomeric proteins and non-sarcomeric proteins.

Previous studies have shown that physical activity significantly influences muscle features such as muscle mass, muscle force, and muscle fiber type (Kanzleiter et al., 2015; Bacurau et al., 2016; Liu et al., 2021). Different from endurance exercise, which can boost muscle strength, aerobic exercise plays a vital role in increasing the aerobic ability of the muscle. Consistent with these findings, aerobic exercise significantly restrained the body weight increase in the exercise group compared with the control group (Figure 2A). Although gastrocnemius mass and the CSA of gastrocnemius fiber in the exercise group did not change when compared with the control group (Figures 2B,D), we observed that the percentage of MyHC I was increased (Figure 2C), suggesting a potential change of “fast” to “slow” muscle fiber. To investigate the molecular mechanism of exercise on muscle functional properties, we focused on lysine acetylation of muscle proteins. Muscle contraction relies on the correct assembly of myofibrils (Wei et al., 2018), which shows alternating light and dark bands under a microscope corresponding to I-bands and A-bands, respectively (Figure 2E). Myofibrils are composed of tandem arrays of basic functional contractile units called the sarcomeres (Hwang and Sykes, 2015; Wei et al., 2018), which are highly ordered structures composed of thin filaments (actin, tropomyosin, and troponin), thick filaments (myosin), and their associated proteins (such as titin, myomesin, and desmin) (Figure 2E). The sarcomeric function is determined by sarcomeric protein isoform expression and PTMs (Hamdani et al., 2008; Russell and Solis, 2021). In our results, sarcomeric protein levels in gastrocnemius muscle showed no change after exercise (Table 1), but their acetylation levels did change (Table 2). Sarcomeric proteins containing Kac sites were mapped to the protein network database, and the global network graph of these interactions is shown in Figure 2F. Exercise can induce acetylation changes at different sites in these proteins, especially in titin and myosin-4. During muscle contraction, the acetylation levels of 36 Kac sites in titin were upregulated or downregulated, including 10 Kac sites in myosin-4 (Figure 2F). Detailed information on these statistically significant acetylated lysine sites is shown in Table 2 and in Huang’s study (Huang et al., 2020). Although the number of sarcomeric proteins identified in the muscle was much less than that in non-sarcomeric proteins, Kac sites of sarcomeric proteins obviously are more abundant (Figure 2F), especially in titin, myh4, and mybpc2, 333, 74, and 17 Kac sites, respectively (Figure 1C). Thus, lysine acetylation is a crucial PTM of muscle sarcomeric proteins during the exercise process.

**TABLE 1:** The expression of sarcomeric proteins in gastrocnemius muscle.

Identified protein relative content	Control group (*n* = 3)			Exercise group (*n* = 3)			Ratio	*P*-value
Obscurin	1.011	0.999	1.009	1.003	1.011	0.986	0.994	4.85E-01
Alpha-actinin-3	1.005	0.986	1.019	1.05	1.039	1.043	1.041	1.65E-02
Myosin light chain 1/3, skeletal muscle isoform	1.105	1.077	1.119	1.017	1.035	1.035	0.938	8.88E-03
Myosin-8	1.05	1.026	1.021	1.093	1.093	1.112	1.065	3.74E-03
Myosin-7B	1.12	0.995	1.047	1.049	1.085	1.099	1.022	5.65E-01
Myosin-7	0.966	0.97	0.95	1.1	1.082	1.076	1.129	1.95E-04
Troponin C, skeletal muscle	0.98	1.008	0.988	1.093	1.084	1.024	1.076	3.06E-02
Tropomyosin alpha-1	0.925	0.949	0.972	1.016	1.045	1.024	1.084	8.22E-03
Tropomyosin alpha-3	0.98	0.943	0.942	0.98	0.956	1.032	1.036	2.50E-01
Tropomyosin beta chain	/							
Troponin I, fast skeletal muscle	0.94	0.909	0.944	0.945	0.939	0.956	1.017	2.67E-01
Troponin T, fast skeletal muscle	0.994	0.937	0.95	0.962	0.957	1.01	1.017	5.40E-01
Desmin	1.1	1.081	1.103	1.036	1.049	1.037	0.951	2.50E-03
Actin, alpha skeletal muscle	1.063	1.095	1.063	1.1	1.103	1.1	1.025	1.26E-01
Actin, alpha cardiac muscle 1	/							
Myosin regulatory light chain 2, skeletal muscle isoform	1.079	1.084	1.089	1.034	1.045	1.052	0.963	2.62E-03
Myosin-4	1.046	1.031	1.035	1.082	1.078	1.095	1.046	2.16E-03
Myosin-1	1.06	1.059	1.074	1.054	1.067	1.062	0.997	6.17E-01
Myosin-binding protein C, fast-type	1.132	1.124	1.136	1.126	1.101	1.114	0.985	1.03E-01
Myomesin-1	1.076	1.046	1.072	1.034	1.043	1.042	0.977	6.32E-02
Myozenin-1	0.85	0.913	0.897	0.923	0.979	0.974	1.081	5.22E-02
Myotilin	1.012	1.018	0.99	1.077	1.056	1.059	1.057	6.12E-03
Myosin-binding protein H	1.106	1.093	1.154	1.189	1.156	1.139	1.039	1.38E-01
Filamin-C	1.008	1.01	1.012	0.996	1	1.009	0.992	1.07E-01

*Protein (peptides) relative content was measured in muscle samples of mice from the control group and exercise group, n = 3 mice/group. Student’s t-test was used in SPSS; there were no significant differences between the two groups. Data about titin were shown in Huang’s study (Huang et al., 2020). Although tropomyosin beta chain and actin alpha cardiac muscle 1 were identified, we did not get its quantitative information. Information about acetylated lysine sites of protein in muscle sarcomere. Modified sequence: identified peptide sequence marked with modification sites’ localization probabilities and amino acid types of all sites is K (Lysine). Protein accession: the identifier of protein in UniProt. Positions: modification site localization in protein. PEP: the maximal posterior error probability for peptides. Ratio: the acetylation fold-change ratio of identified lysine sites. Score: the −10 logarithmic probability of observing the given number of matches or more by chance. Student’s t-test was used in SPSS. Data about titin were shown in Huang’s study (Huang et al., 2020).*

**TABLE 2:** Alterations of lysine acetylation sites in proteins of muscle sarcomere during exercise.

Protein accession	Position	Protein description	Gene name	PEP	Score	Modified sequence	Ratio	*P*-value
A2AAJ9	941	Obscurin	Obscn	3.08E-26	126.07	ADAGEYSCEAGGQK(1)LSFR	1.221	3.10E-02
O88990	411	Alpha-actinin-3	Actn3	2.77E-06	93.345	LQHLAEK(1)FQQK	0.818	3.07E-02
P05977	31	Myosin light chain 1/3, skeletal muscle isoform	Myl1	1.89E-06	111.79	EEK(1)IDLSAIK	0.7	5.92E-03
P13542	568	Myosin-8	Myh8	0.00434743	90.15	SNNFQK(1)PK	1.232	3.66E-02
P20801	85	Troponin C, skeletal muscle	Tnnc2	0.029179	51.066	QMK(1)EDAK	0.787	4.80E-03
P31001	43	Desmin	Des	0.00134246	65.467	AGFGTK(1)GSSSSMTSR	2.096	7.66E-03
P68134	330	Actin, alpha skeletal muscle	Acta1	0.00851098	66.056	IK(1)IIAPPER	0.71	1.55E-02
P68134	293	Actin, alpha skeletal muscle	Acta1	4.69E-21	88.239	K(1)DLYANNVMSGGTTMYPGIADR	1.203	7.03E-04
P97457	166	Myosin regulatory light chain 2, skeletal muscle isoform	Mylpf	8.53E-65	178.51	NICYVITHGDAK(1)DQE	0.613	2.82E-03
Q5SX39	955	Myosin-4	Myh4	3.19E-41	168.3	LEDECSELK(1)K	0.769	8.04E-03
Q5SX39	453	Myosin-4	Myh4	4.61E-06	94.547	INQQLDTK(1)QPR	0.813	4.84E-03
Q5SX39	1448	Myosin-4	Myh4	2.62E-09	111.12	SNAACAALDK(1)K	0.796	5.14E-03
Q5SX39	761	Myosin-4	Myh4	2.30E-48	149.73	LLGSIDIDHTQYK(1)FGHTK	0.82	4.18E-02
Q5SX39	914	Myosin-4	Myh4	0.00226055	100.88	CDQLIK(1)TK	1.423	2.32E-03
Q5SX39	59	Myosin-4	Myh4	0.0342729	56.404	EGGK(1)VTAK	1.548	4.31E-02
Q5SX39	1420	Myosin-4	Myh4	0.00346135	96.342	CASLEK(1)TK	1.422	1.65E-02
Q5SX39	972	Myosin-4	Myh4	0.00392593	93.096	EK(1)HATENK	1.384	2.84E-03
Q5SX39	562	Myosin-4	Myh4	6.48E-49	152.37	LYEQHLGK(1)SNNFQKPK	0.826	7.96E-03
Q5SX39	1758	Myosin-4	Myh4	0.0170478	86.136	NAEEK(1)AK	1.311	5.27E-03
Q5SX40	1451	Myosin-1	Myh1	1.01E-13	131.66	TNAACAALDK(1)K	0.75	4.97E-04
Q5SX40	1535	Myosin-1	Myh1	0.00205512	83.206	IHELEK(1)IK	1.3	7.56E-05
Q5XKE0	387	Myosin-binding protein C, fast-type	Mybpc2	0.030363	71.379	EDSYK(1)AR	0.612	7.40E-04
Q5XKE0	88	Myosin-binding protein C, fast-type	Mybpc2	0.00599854	69.864	GK(1)WQELGSK	0.698	3.18E-03
Q5XKE0	304	Myosin-binding protein C, fast-type	Mybpc2	9.19E-05	112.13	YVFENVGK(1)K	0.563	2.76E-03
Q5XKE0	1017	Myosin-binding protein C, fast-type	Mybpc2	1.21E-30	125.45	IFSENICGLSDSPGVSK(1)NTAR	1.642	2.17E-02
Q62234	237	Myomesin-1	Myom1	1.29E-14	127.02	K(1)TLEETQTYHGK	0.771	4.41E-02
Q8VHX6	735	Filamin-C	Flnc	6.84E-07	112.94	CSYVPTK(1)PIK	1.335	2.26E-03

*The annotations are the same as above (Table 1). There were no significant differences between the two groups. Information about acetylated lysine sites of three enzymes. The annotations are the same as above (Table 2).*

### Enrichment Analysis of the Lysine Acetylation Level of Proteins

**FIGURE 3:**
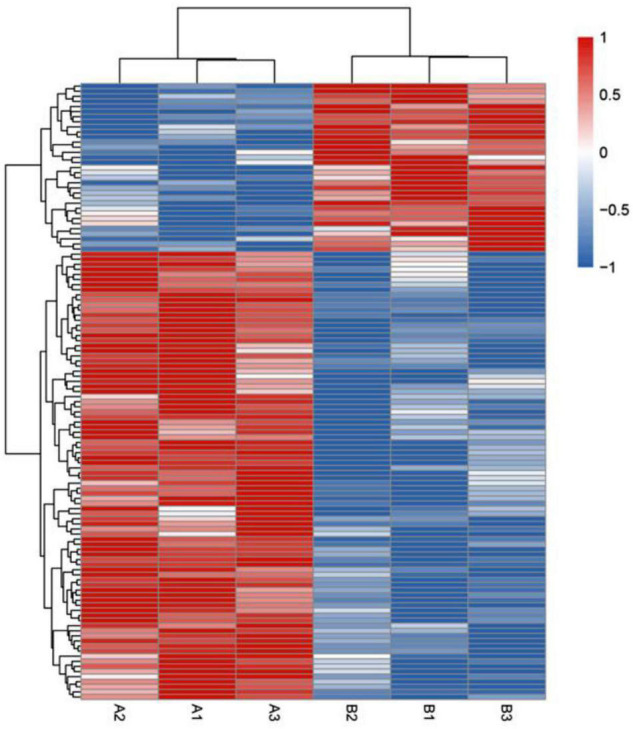
Heatmap showing hierarchical cluster analysis of lysine-acetylated proteins in control groups (A1–A3) and exercise groups (B1–B3). The Euclidean distance was used as the distance metric, and the clustering criteria were complete. The heatmap includes an accompanying supplemental table with the list of the proteins used and their fold change (supplemental “DEP heatmap”).

**FIGURE 4:**
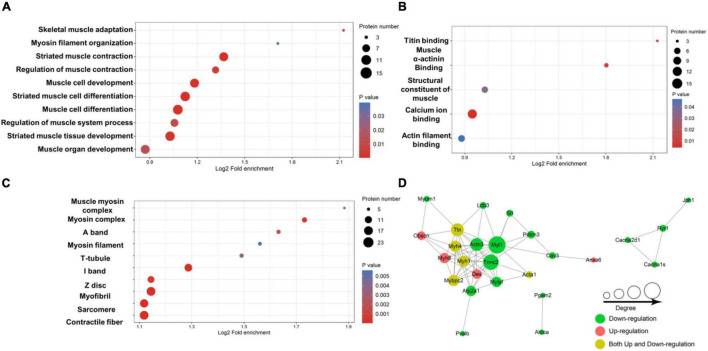
Enrichment analysis of the lysine-acetylated proteins. Enrichment based on GO annotation in terms of biological process **(A)**, molecular function **(B)**, and cellular component **(C)**. **(D)** Protein–protein interaction network for acetylated proteins. The nodes represent proteins, and their colors represent the upregulation or downregulation of these proteins; green means downregulation (change ratio < 0.83), red means upregulation (change ratio > 1.20), and yellow means both upregulation and downregulation. The size of each node represents the number of directed edges.

To further test the role of lysine acetylation in regulating muscle biological functions, we performed an enrichment analysis. Differential lysine acetylation levels of proteins related to muscle contraction are shown in Figure 3. The site-specific heatmap analysis revealed a clear boundary between the control group and the exercise group, indicating that exercise induced significant changes in the acetylation levels of these proteins, which could be upregulated or downregulated (Figure 3). GO enrichment was performed to better understand the specific role of lysine-acetylated proteins in muscle. A bubble plot of GO enrichment of proteins corresponding to lysine acetylation sites is shown in Figures 4A–C. GO enrichment of biological processes revealed significant protein enrichment in terms related to skeletal muscle adaptation, striated muscle contraction, regulation of muscle contraction, muscle cell development, and others (*p* < 0.05; Figure 4A). The results of molecular function analysis also showed that proteins related to titin binding, muscle alpha-actinin binding, and calcium ion binding were significantly enriched (*p* < 0.05; Figure 4B). Cellular component analysis indicated that acetylated proteins were enriched in the muscle myosin complex, I-band, sarcomere, myofibril, and others (*p* < 0.05; Figure 4C). In addition, a PPI network containing 26 nodes and 67 edges was constructed (Figure 4D). In this study, the PPI network was constructed of acetylated proteins identified as nodes and connected with each other. Many proteins in the PPI network contained numerous Kac sites. In summary, these data suggest that exercise-induced lysine acetylation is critical in muscle protein contraction and structure.

### Molecular Docking Analysis of Enzymes

**FIGURE 5:**
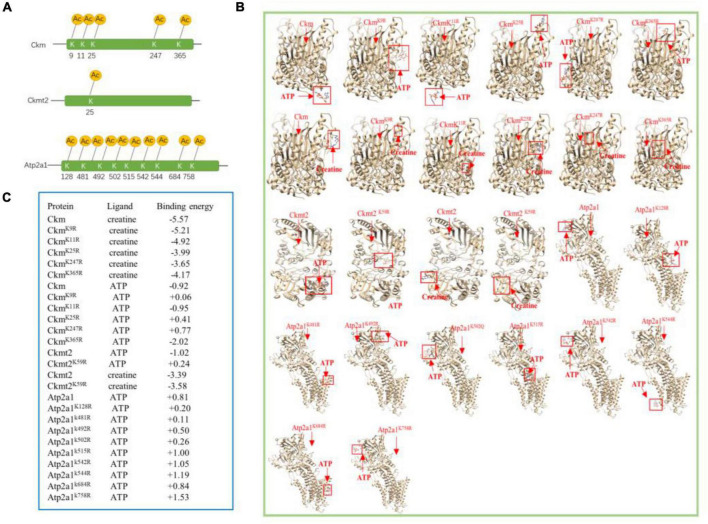
Molecular docking analysis of enzymes. **(A)** Localizations of the multiacetylated sites in three enzymes that were identified with 5, 1, and 9 acetylated lysine residues. **(B)** AutoDock 4.2 was used to predict docking interactions of proteins and ligands. The binding positions are highlighted by red boxes. Ckm and Ckmt2 both have two ligands, and Atp2a1 has one ligand. **(C)** Docking results of the three enzymes.

Extensive reversible lysine acetylation of metabolic enzymes occurs in both eukaryotes and prokaryotes (Zhao et al., 2010; Nakayasu et al., 2017), which could influence energy balance. Muscle is a high-energy-consuming tissue, so lysine acetylation will control its energy homeostasis (Menzies and Auwerx, 2013). In our study, we identified multiple Kac sites on three enzymes involved in muscle contraction, namely, creatine kinase M-type (Ckm), creatine kinase S-type (Ckmt2), and sarcoplasmic/endoplasmic reticulum calcium ATPase 1 (Atp2a1). There were 22, 8, and 20 Kac sites in Ckm, Ckmt2, and Atp2a1, respectively. There was no alteration in the protein abundance levels of the three enzymes (Table 3), whereas their acetylation levels did change. Interestingly, all Kac sites of the three proteins were deacetylated rather than acetylated. After exercise, the numbers of deacetylated sites of Ckm, Ckmt2, and Atp2a1 were 5, 1, and 10, respectively (Figure 5A and Table 4). Molecular docking is a well-established approach for predicting binding conformations of ligands in active or binding sites of relevant protein targets (Bafna et al., 2020). The catalytic consequences of K9, K11, K25, K247, and K365 deacetylation in Ckm, as well as Ckmt2 and Atp2a1, were evaluated using lysine-to-arginine (K-to-R) mutants *in vitro*, which mimic deacetylated lysine residues. The docking results showed that deacetylation significantly changed the substrate binding energy and binding regions (Figures 5B,C). For instance, the binding energy between Ckm and creatine was −5.57 kcal/mol, whereas the binding energy between Ckm^K9R^ and creatine was −5.21 kcal/mol, and the binding active site changed as well. These data suggest that deacetylation impeded two-molecule interactions and then influenced enzyme activity.

**TABLE 3:** The expression of three enzymes in the gastrocnemius muscle.

Identified protein relative content	Control group (*n* = 3)			Exercise group (*n* = 3)			Ratio	*P*-value
Creatine kinase M-type 0.976	0.973	0.976	0.976	0.977	0.982	0.991	1.009	1.19E-01
Creatine kinase S-type, mitochondrial	0.978	0.969	0.963	1.021	1.009	1.032	1.052	3.00E-03
Sarcoplasmic/endoplasmic reticulum calcium ATPase 1	0.986	0.983	0.999	0.969	0.993	0.98	1.043	5.72E-03

**TABLE 4:** Deacetylated sites of Ckm, Ckmt2, and Atp2a1 after exercise.

Protein accession	Position	Protein description	Gene name	PEP	Score	Modified sequence	Ratio	*P*-value
P07310	365	Creatine kinase M-type	Ckm	0.000163	113.62	LMVEMEK(1)K	0.758	4.20E-03
P07310	11	Creatine kinase M-type	Ckm	3.58E-80	189.87	FK(1)LNYKPQEEYPDLSK	0.536	6.21E-04
P07310	25	Creatine kinase M-type	Ckm	2.20E-123	205.96	LNYKPQEEYPDLSK(1)HNNHMAK	0.759	2.95E-04
P07310	9	Creatine kinase M-type	Ckm	4.46E-34	164.64	PFGNTHNK(1)FK	0.649	2.84E-03
P07310	247	Creatine kinase M-type	Ckm	0.000281	104.22	GGNMK(1)EVFRR	0.747	4.52E-03
Q6P8J7	59	Creatine kinase S-type, mitochondrial	Ckmt2	4.68E-25	123.4	K(1)HNNCMAECLTPTIYAK	0.751	6.17E-05
Q8R429	128	Sarcoplasmic/endoplasmic reticulum calcium ATPase 1	Atp2a1	0.001152	65.179	EYEPEMGK(1)VYR	0.558	1.46E-03
Q8R429	542	Sarcoplasmic/endoplasmic reticulum calcium ATPase 1	Atp2a1	0.000138	85.813	VPLTGPVK(1)EK	0.665	1.24E-03
Q8R429	492	Sarcoplasmic/endoplasmic reticulum calcium ATPase 1	Atp2a1	2.71E-09	119.25	K(1)SMSVYCSPAK	0.651	2.04E-03
Q8R429	502	Sarcoplasmic/endoplasmic reticulum calcium ATPase 1	Atp2a1	1.03E-15	117.48	SMSVYCSPAK(1)SSR	0.582	1.81E-04
Q8R429	758	Sarcoplasmic/endoplasmic reticulum calcium ATPase 1	Atp2a1	5.39E-10	121.41	AIYNNMK(1)QFIR	0.813	1.08E-02
Q8R429	684	Sarcoplasmic/endoplasmic reticulum calcium ATPase 1	Atp2a1	0.000925	105.98	VEPSHK(1)SK	0.671	4.82E-04
Q8R429	481	Sarcoplasmic/endoplasmic reticulum calcium ATPase 1	Atp2a1	3.25E-05	113.61	K(1)EFTLEFSR	0.757	2.64E-03
Q8R429	544	Sarcoplasmic/endoplasmic reticulum calcium ATPase 1	Atp2a1	0.00178	102.52	EK(1)IMSVIK	0.767	1.16E-02
Q8R429	515	Sarcoplasmic/endoplasmic reticulum calcium ATPase 1	Atp2a1	8.72E-13	106.14	MFVK(1)GAPEGVIDR	0.68	1.36E-03

In short, a particular map of lysine acetylation sites from the skeletal muscle of mice was produced by high-resolution tandem mass spectrometry. Our results revealed that exercise-induced acetylation may play a crucial role in skeletal muscle protein binding free energy, at least to some extent.

## Discussion

In our study, the body weights of the mice in these two groups all increased significantly, and exercise slowed the rate of weight gain (Figure 2A). We did not find a significant difference in the gastrocnemius mass or the CSA of the gastrocnemius fiber between the two groups (Figures 2B,D), but the expression levels of MyHC genes were changed, suggesting that the aerobic training program may be able to switch the fiber from the fast- to the slow-twitch type (Figure 2C). While the results of Oil Red O staining revealed that there was little effect of aerobic exercise on the CSA of muscle, exercise obviously boosted lipid deposition in muscle compared with the sedentary group (Figure 2D), suggesting that physical training increases the energy reserves of muscle. Exercise remains beneficial for the musculoskeletal system, which is a very complex physiological process that requires an appropriate regulatory network (Egan and Zierath, 2013; Hoffman et al., 2015). Protein lysine acetylation/deacetylation in skeletal muscle could be a mediator of this network in mice during exercise. We used the acetylproteomics approach to quantify and map acetyl sites after aerobic exercise in mice. Lundby found that the muscle was the most acetylation-accessible tissue in 16 rat tissues (Lundby et al., 2012), and we also identified large numbers of Kac sites in mouse muscle proteins (Figure 1B). By comparing these two studies, we found that protein acetylation is widespread regardless of species, and the abundance levels of protein lysine acetylation in human, rat, and mouse muscle tissue are similar (Figure 1D). Interestingly, dramatic changes in the acetylation status of numerous cytoplasmic proteins were observed, but the expression levels of KATs and HDACs detected in our proteomic analysis were similar in the two groups (Supplementary Table 1). It is possible that other KATs and HDACs we did not identify in the mass spectrometry are involved in the progress.

In our results, more than half of the proteins (53.70%) had one Kac site and only 15 proteins (2.16%) contained >15 Kac sites (Figure 1C). The importance of acetylation modification is well appreciated in gene regulation, but its regulatory implications extend beyond nuclear histone modifications. We found that sarcomeric proteins accounted for a huge percentage of numerous-site proteins, especially titin (333 sites) and myosin-4 (74 sites). Protein acetylation occurs widely in sarcomeres (Gupta et al., 2008; Samant et al., 2015; Lin et al., 2016) and significantly influences sarcomere function. Gupta provided evidence that the reversible acetylation of sarcomeres plays a role in the regulation of myofilament contractile activity (Gupta et al., 2008). In our study, there were 25 sarcomeric proteins containing Kac sites, which were distributed on thin filaments, thick filaments, or *Z*-disks. After exercise, the Kac sites of 14 proteins were acetylated or deacetylated (Figure 2F). Thus, exercise triggered protein acetylation or deacetylation in sarcomeres, especially in titin and myosin-4. More importantly, the mean lysine acetylation level of sarcomeric proteins was higher than that of their non-sarcomeric counterparts (Figure 2F). These findings suggested that acetylation modification plays an important role in sarcomeric proteins, which might contribute to muscle contraction. However, the effects of sarcomeric protein acetylation on myofilament contractile activity during exercise remain unclear.

To further understand the potential effect of protein acetylation on muscle structure and function, we performed a bioinformatics analysis. The site-specific heatmap showed that exercise-regulated lysine-acetylated proteins are involved in muscle contraction (Figure 3). According to GO enrichment analysis, the results classification for biological process, molecular function, and cellular component highlighted that these acetylated/deacetylated proteins correspond to different muscle structures or functions (Figures 4A–C). In addition, the protein network analysis indicated that the physiological interactions of these proteins identified in GO enrichment were likely to contribute to their cooperation in muscle (Figure 4D).

Since lysine residues of enzymes are always present around their active sites, protein acetylation influences the catalytic activity of enzymes involved in cellular metabolism (Hernandez-Saavedra et al., 2019; Wang et al., 2019; Min et al., 2020). Lundby reported that multiple enzymes involved in ATP generation are hyperacetylated in skeletal muscle mitochondria (Lundby et al., 2012), and protein acetylation of some enzymes was thought to be involved in impaired fatty acid oxidation and exercise intolerance (Tsuda et al., 2018). In addition, the enzymatic activity of many mitochondrial protein bacterial orthologs is regulated by lysine acetylation (Wang et al., 2010). Interestingly, we found that there were three enzymes directly linked with muscle energy regulation, namely, Ckm, Ckmt2, and Atp2a1. Then, we used AutoDock software to simulate the effects of lysine acetylation on these metabolic enzymes. Compared with wild-type enzymes, mimetic mutants appear to have different substrate-binding energies and binding positions (Figures 5B,C). Consistent with a previous study (Walker et al., 2021), acetylation of Ckm disrupted salt bridge formations between Ckm monomers, including K9, K11, and K25, in this study. In addition, Gorski found that acetylation at K492 played a critical role in regulating SERCA2a (sarcoplasmic/endoplasmic reticulum Ca^2+^ ATPase 2a) activity in mice (Gorski et al., 2019). Therefore, the deacetylation of these lysine sites was likely to affect the catalytic reaction of Ckm, Ckmt2, and Atp2a1, which may be associated with the impact of acetylation on the dimer formation of Ckm and Ckmt2 (Walker et al., 2021), although site-specific contributions were indistinct.

It is widely accepted that aerobic exercise can increase exercise capacity, such as muscle oxidative capacity, muscle contraction, and muscle endurance (Overmyer et al., 2015; de Oliveira et al., 2017; Huang et al., 2020). Furthermore, muscle protein acetylation levels also significantly changed after 6 weeks of aerobic exercise. Previously, Gupta reported that reversible acetylation of sarcomeric proteins played a role in regulating myofilament contractile activity (Gupta et al., 2008). Similarly, lysine acetylation of cardiac myosin heavy chain isoforms modulates their enzymatic and motor activity (Samant et al., 2015). Some researchers have also demonstrated that altering the muscle structure and function by acetylation of existing sarcomeric proteins can optimize energy usage (Russell and Solis, 2021). With these observations, it is reasonable to speculate that lysine acetylation is associated with alterations in muscle protein binding free energy during exercise.

We showed that exercise changes muscle protein acetylation in mice, which serves as a reference for further Kac studies in human. In these future clinical and mechanistic studies, verification of the correlation between protein acetylation and skeletal muscle physiology or exercise performance should be taken into account, which will improve our understanding of the molecular response to regular exercise. Ongoing acetylproteomics studies will provide a theoretical basis for exercise prescription. In addition, novel approaches targeting the acetylation of skeletal muscle proteins, especially sarcomeric proteins, would provide important clues for the development of an effective intervention for muscle atrophy with aging. In any case, these results from our study still need to be confirmed in clinical trials.

However, there are some limitations to this study. First, the effects of acetylation on the mechanical parameters of sarcomere proteins remain unknown. Then, we did not perform the same experiments on female mice or old mice, which may not be replicated because sex and age are also important biological variables. In addition, other exercise parameters (such as session length, frequency, and intensity) were not tested in our study. Finally, the impact of deacetylation on enzyme activity requires further experimental verification.

## Conclusion

Our data strongly suggested that the regulatory scope of exercise-induced lysine acetylation is widespread in skeletal muscle proteins. Exploring the mechanisms of exercise-induced acetylation changes in skeletal muscle can help develop scientific exercise prescriptions. Overall, skeletal muscle exhibits extensive metabolic and energetic remodeling during exercise, and exercise-induced lysine acetylation appears to be a crucial mediator of skeletal muscle proteins.

## Data Availability Statement

The mass spectrometry proteomics data have been deposited to the ProteomeXchange Consortium (http://proteomecentral.proteomexchange.org) via the iProX partner repository (Ma et al., 2019) with the dataset identifier PXD032660.

## Ethics Statement

The animal study was reviewed and approved by the SYXK-2019-0004.

## Author Contributions

DL prepared the figures and drafted the manuscript. LF and YN edited and revised the manuscript. CC, SH, SL, LF, and YN approved the final version of the manuscript. All authors contributed to the article and approved the submitted version.

## Conflict of Interest

The authors declare that the research was conducted in the absence of any commercial or financial relationships that could be construed as a potential conflict of interest.

## Publisher’s Note

All claims expressed in this article are solely those of the authors and do not necessarily represent those of their affiliated organizations, or those of the publisher, the editors and the reviewers. Any product that may be evaluated in this article, or claim that may be made by its manufacturer, is not guaranteed or endorsed by the publisher.

## Footnotes

1
http://www.ebi.ac.uk/GOA

2
https://www.string-db.org

3
https://www.uniprot.org

4
https://pubchem.ncbi.nlm.nih.gov

## Abbreviations

Atp2a1: sarcoplasmic/endoplasmic reticulum calcium ATPase 1
Ckm: creatine kinase M-type
Ckmt2: creatine kinase S-type
DTT: dithiothreitol
FDR: false discovery rate
GO: Gene Ontology
Kac: lysine-acetylated
NAM: nicotinamide
PTM: post-translational modification
TFA: trifluoroacetic acid
TSA: trichostatin.
