# Supplementary material for: Alterations of Lysine Acetylation Profile in Murine Skeletal Muscles Upon Exercise
Source: Front Aging Neurosci. 2022 May 3;14:859313. doi: 10.3389/fnagi.2022.859313 (PMC9110802; doi:10.3389/fnagi.2022.859313)
Supplement: Supplementary file 1 [file Table_1.DOCX]

Table S1. The expression of Acetyltransferase and deacetylase in gastrocnemius muscle of mice. Protein (peptides) relative content was measured in muscle samples of mice from control group and exercise group, n=3 mice/group. Ratio: the acetylation fold change ratio of identified lysine sites. Student’s t-test was used in SPSS. Although Histone deacetylase 4, N-alpha-acetyltransferase 35, N-alpha-acetyltransferase 30 and Elongator complex protein 3 were identified, we didn’t get its quantitative information.

| Identified Protein  Relative Content | Control Group(n=3) | Exercise Group(n=3) | Ratio | *P* value |
| --- | --- | --- | --- | --- |
| N-alpha-acetyltransferase 20 | 0.848 0.908 1.073 | 1.086 1.052 0.94 | 1.088 | 3.49E-01 |
| Histone deacetylase 2 | 0.962 0.865 1.02 | 0.975 0.989 1.013 | 1.046 | 3.98E-01 |
| Histone deacetylase 4 | / | | | |
| N-alpha-acetyltransferase 50 | 0.992 0.927 0.948 | 1.013 1.021 1.029 | 1.068 | 3.13E-02 |
| N-alpha-acetyltransferase 35 | / | | | |
| N-alpha-acetyltransferase 15 | 0.972 1.035 1.02 | 0.982 1.043 0.986 | 0.995 | 8.54E-01 |
| N-alpha-acetyltransferase 25 | 0.923 0.934 0.875 | 1.088 0.986 1.099 | 1.161 | 2.05E-02 |
| N-alpha-acetyltransferase 30 | / | | | |
| NAD-dependent protein deacylase sirtuin-5 | 0.927 0.957 1.018 | 1.046 1.085 0.972 | 1.069 | 1.91E-01 |
| NAD-dependent protein deacetylase sirtuin-3 | 0.9 1.039 0.89 | 1.105 0.991 1.034 | 1.106 | 1.57E-01 |
| NAD-dependent protein deacetylase sirtuin-2 | 1.023 1.01 1.016 | 0.944 0.96 0.959 | 0.939 | 6.62E-04 |
| Elongator complex protein 3 | / | | | |
| N-alpha-acetyltransferase 10 | 0.797 0.896 0.88 | 1.093 0.896 0.953 | 1.143 | 1.29E-01 |
