# Supplementary figures and images for: Alterations of Lysine Acetylation Profile in Murine Skeletal Muscles Upon Exercise
Source: Front Aging Neurosci. 2022 May 3;14:859313. doi: 10.3389/fnagi.2022.859313 (PMC9110802; doi:10.3389/fnagi.2022.859313)

| Raw file    | Scan number | Mass analyzer | Score  | m/z      | Proteins |
|-------------|-------------|---------------|--------|----------|----------|
| F8184TPAc_1 | 8854        | FTMS          | 103.88 | 616.8066 | P07310   |

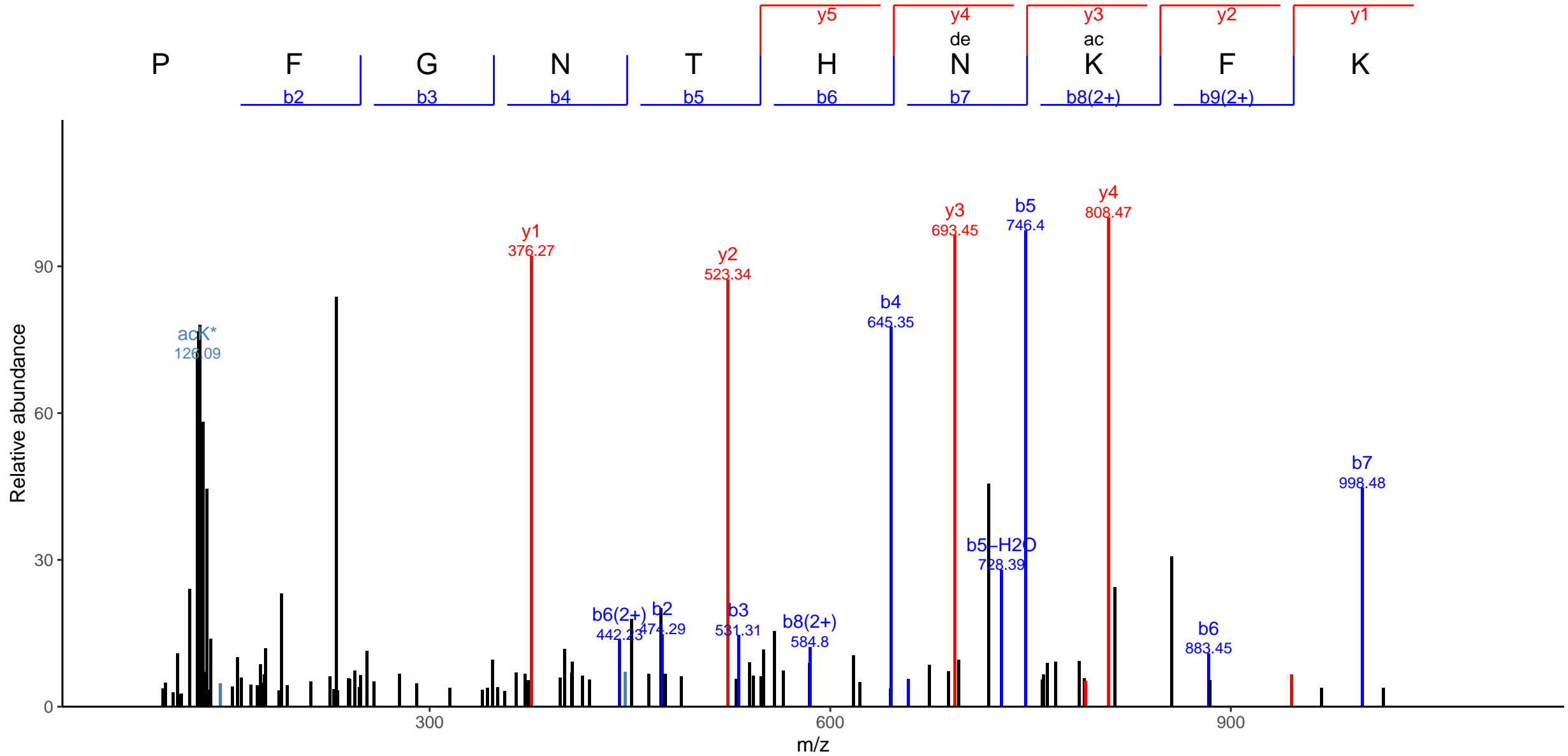

Supplement: Supplementary file 3 [file Image_1.pdf]

| Raw file    | Scan number | Mass analyzer | Score  | m/z      | Proteins             |
|-------------|-------------|---------------|--------|----------|----------------------|
| F8184TPAc_4 | 7432        | FTMS          | 142.38 | 710.8531 | P13542;Q5SX39;Q5SX40 |

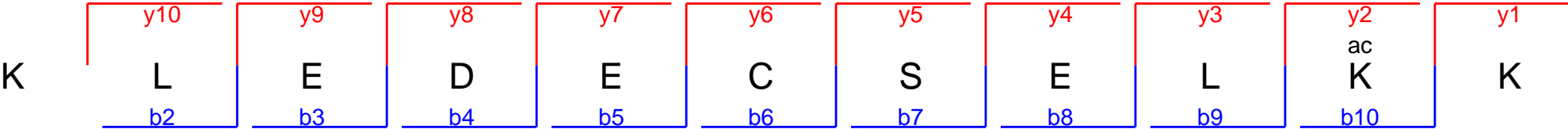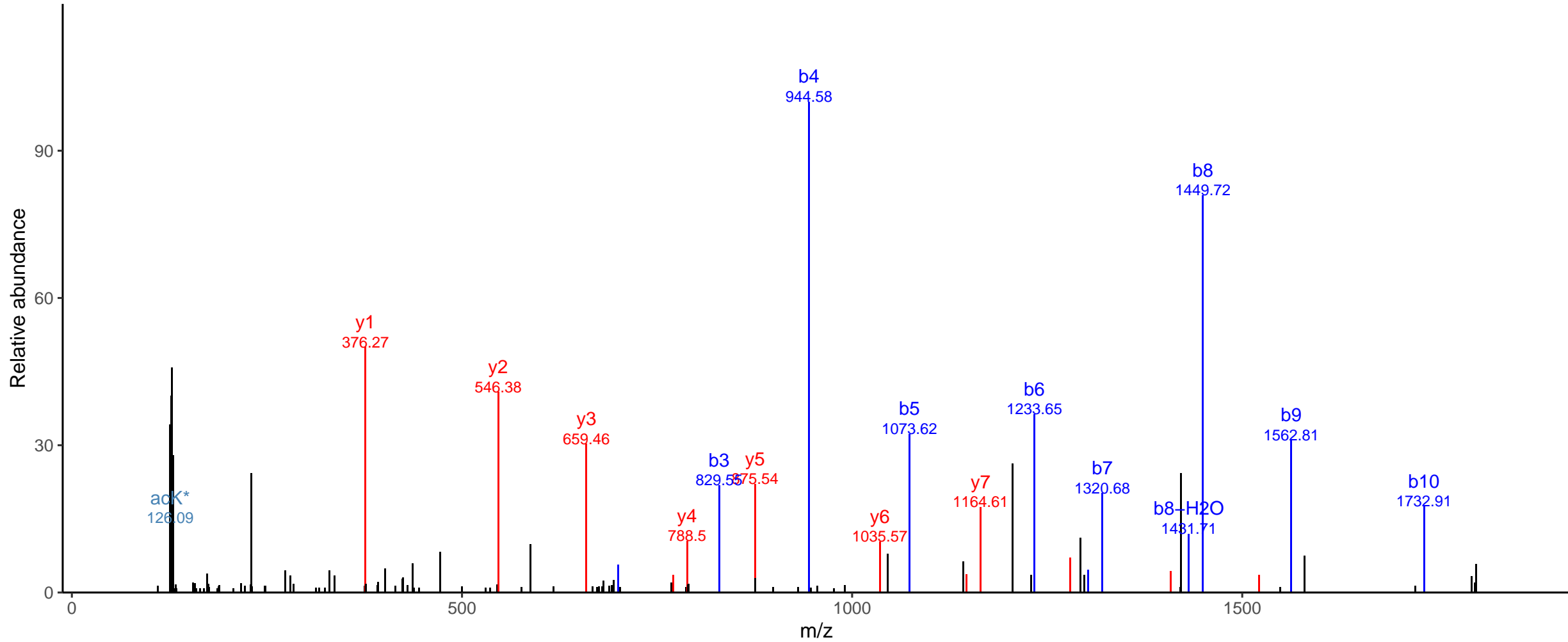

Supplement: Supplementary file 4 [file Image_2.pdf]

| Raw file    | Scan number | Mass analyzer | Score  | m/z      | Proteins |
|-------------|-------------|---------------|--------|----------|----------|
| F8184TPAc_4 | 16431       | FTMS          | 78.814 | 832.4454 | A2ASS6   |

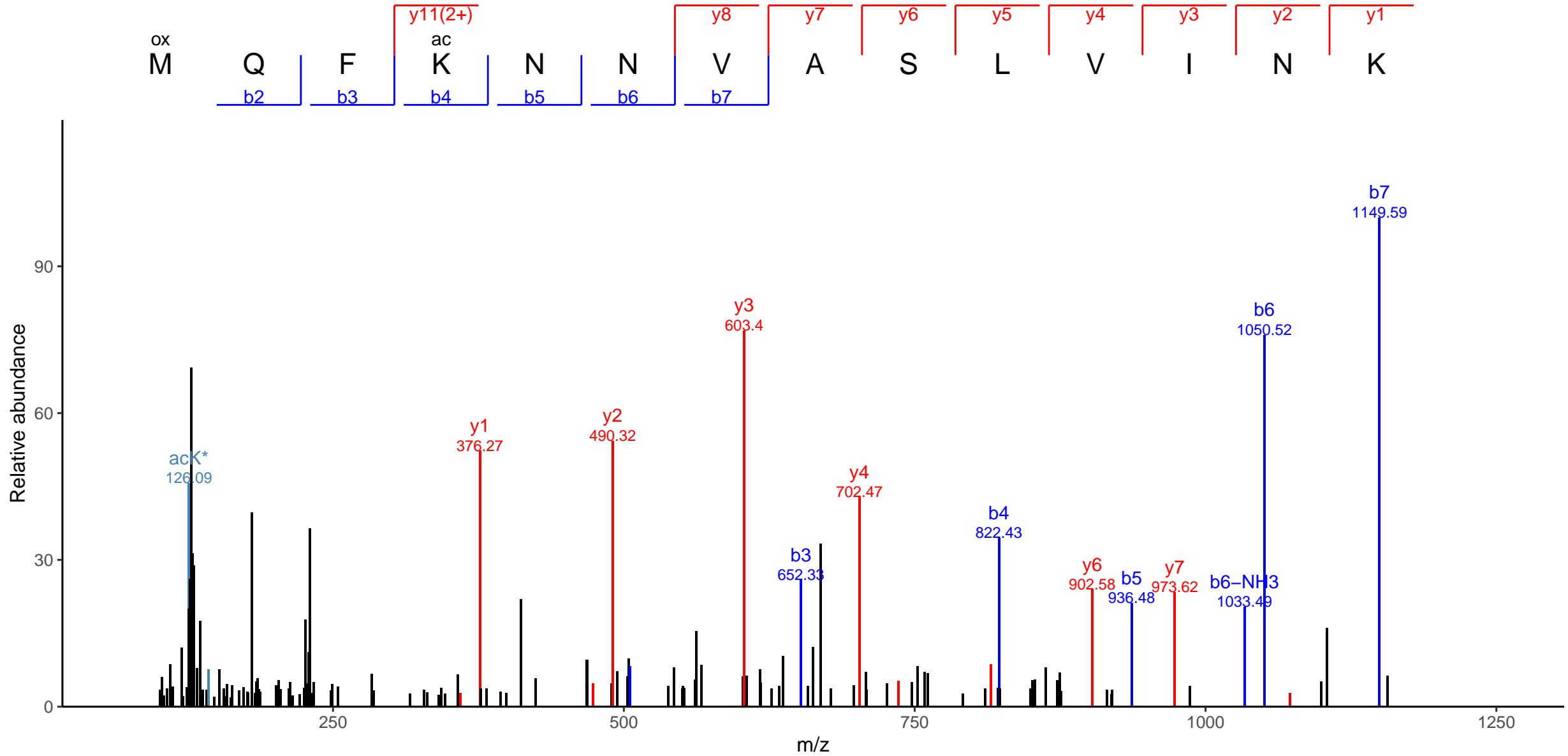

Supplement: Supplementary file 5 [file Image_3.pdf]

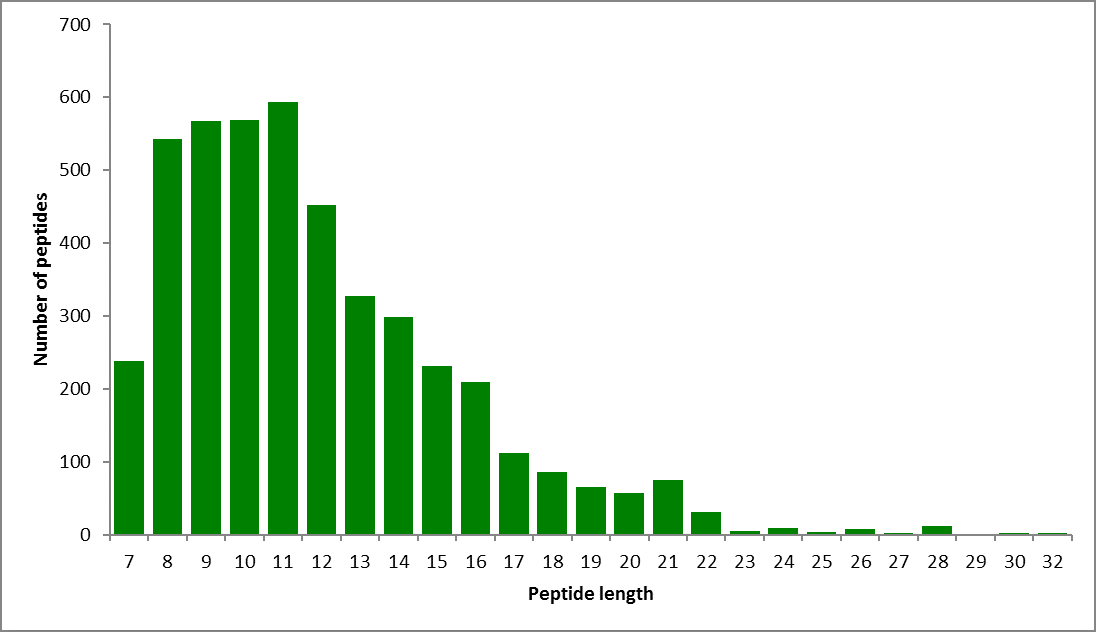

Supplement: Supplementary file 6 [file Image_4.png]

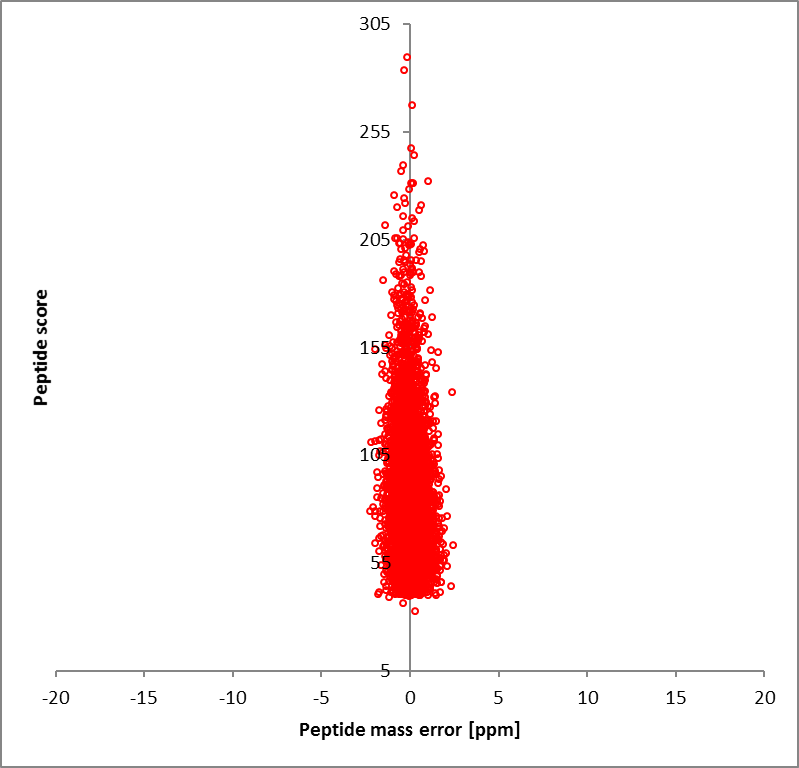

Supplement: Supplementary file 7 [file Image_5.png]
